# Supplementary figures and images for: Object knowledge representation in the human visual cortex requires a connection with the language system
Source: PLoS Biol. 2025 May 20;23(5):e3003161. doi: 10.1371/journal.pbio.3003161 (PMC12091770; doi:10.1371/journal.pbio.3003161)

**A** Raw object-color representation

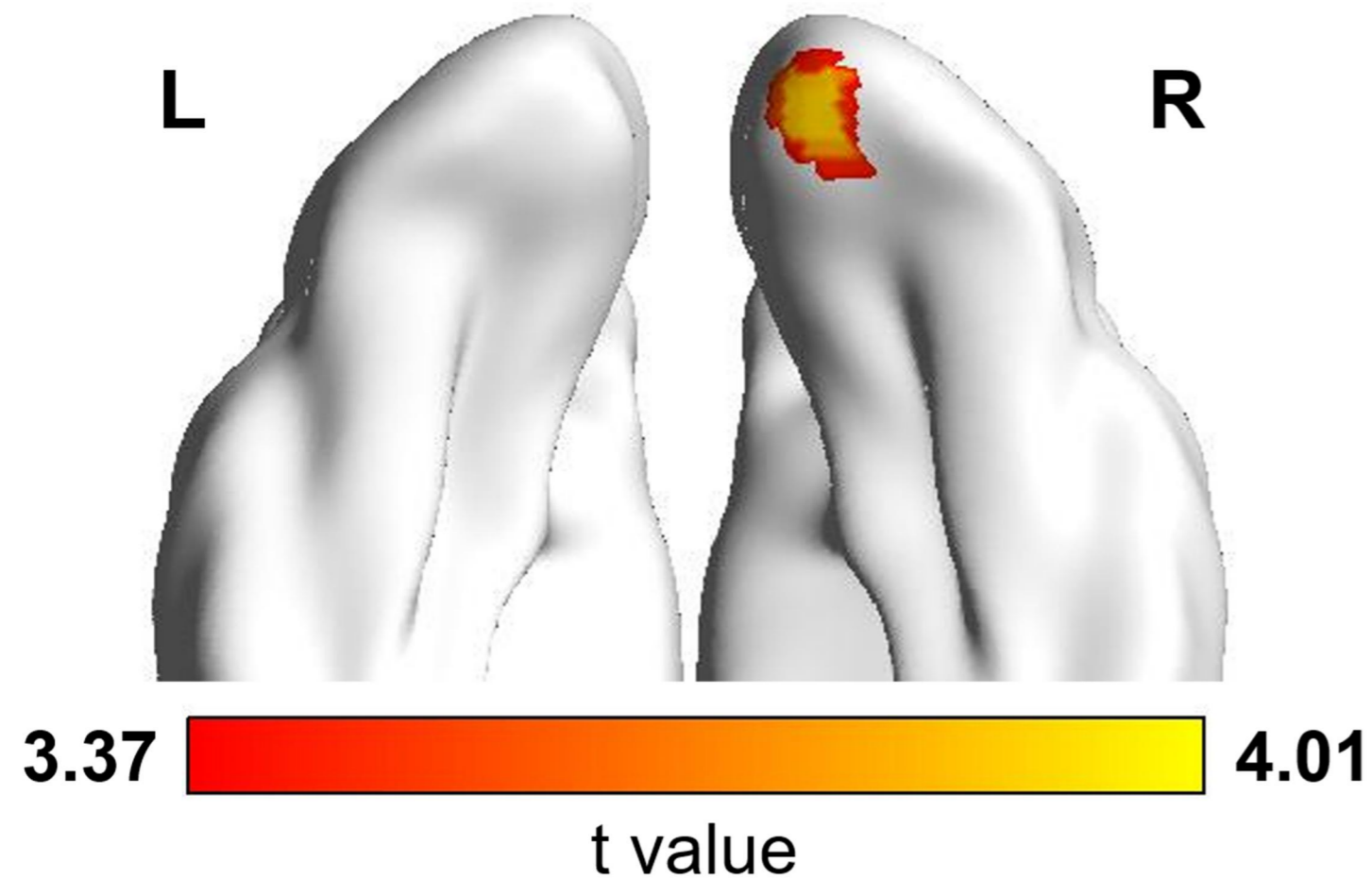

**B** Unique object-color representation

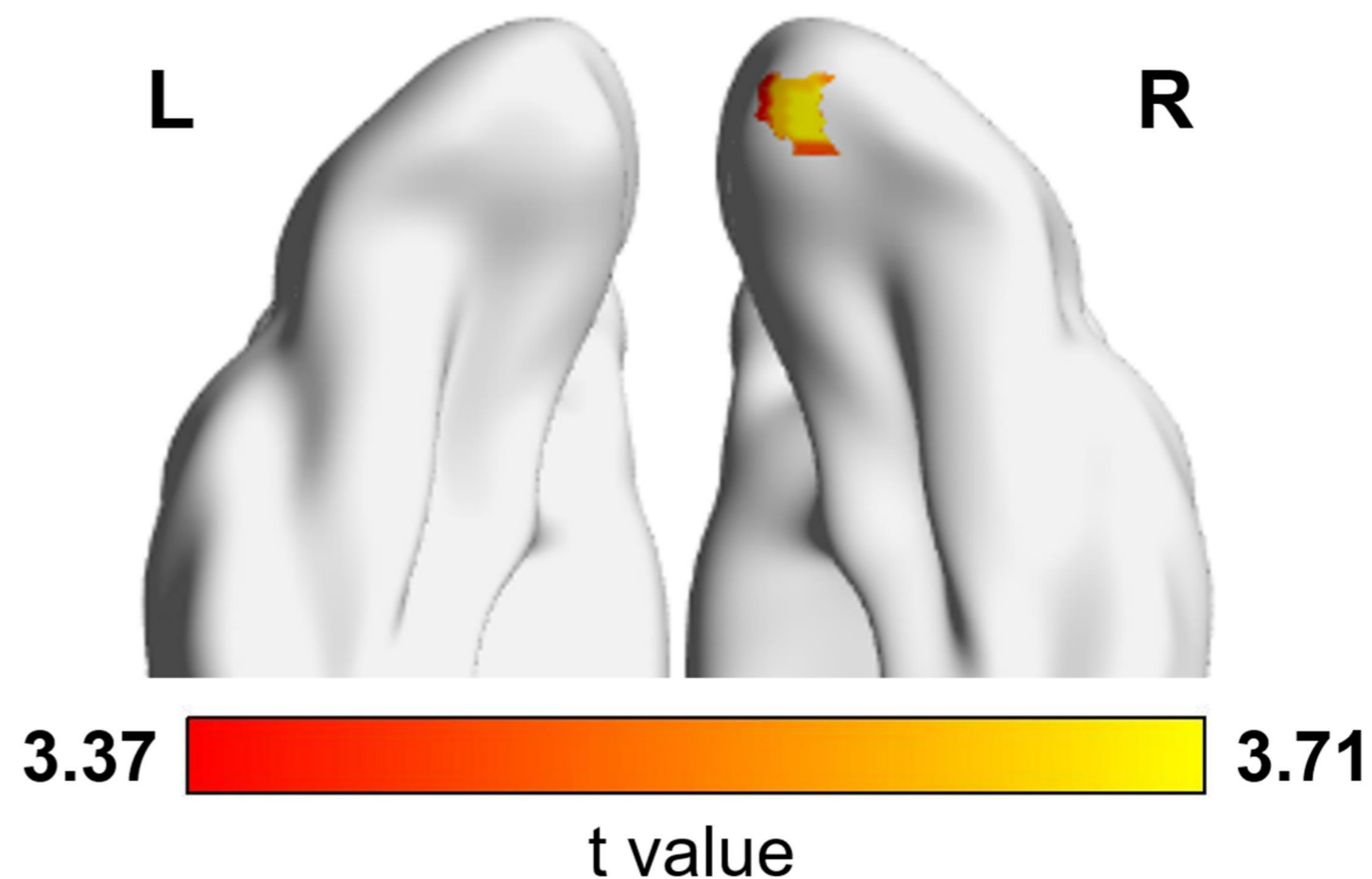

Supplement: S1 Fig — (A) Raw object-color representation (correlating neural RDMs with the behavioral object color similarity RDM) peaked in the right lingual gyrus (peak MNI xyz: 16, −88, −8; peak t = 4.01; 35 voxels). (B) Unique object-color representation, after controlling for low-level visual RDM, shape RDM, and general semantic RDM, showed similar anatomical locations (peak MNI xyz: 16, −90, −8; peak t = 3.71; 19 voxels). Brain results were visualized using BrainNet Viewer (version 1.7; https://www.nitrc.org/projects/bnv/; RRID: SCR_009446). (PDF) [file pbio.3003161.s001.pdf]

### Healthy controls (n=32)

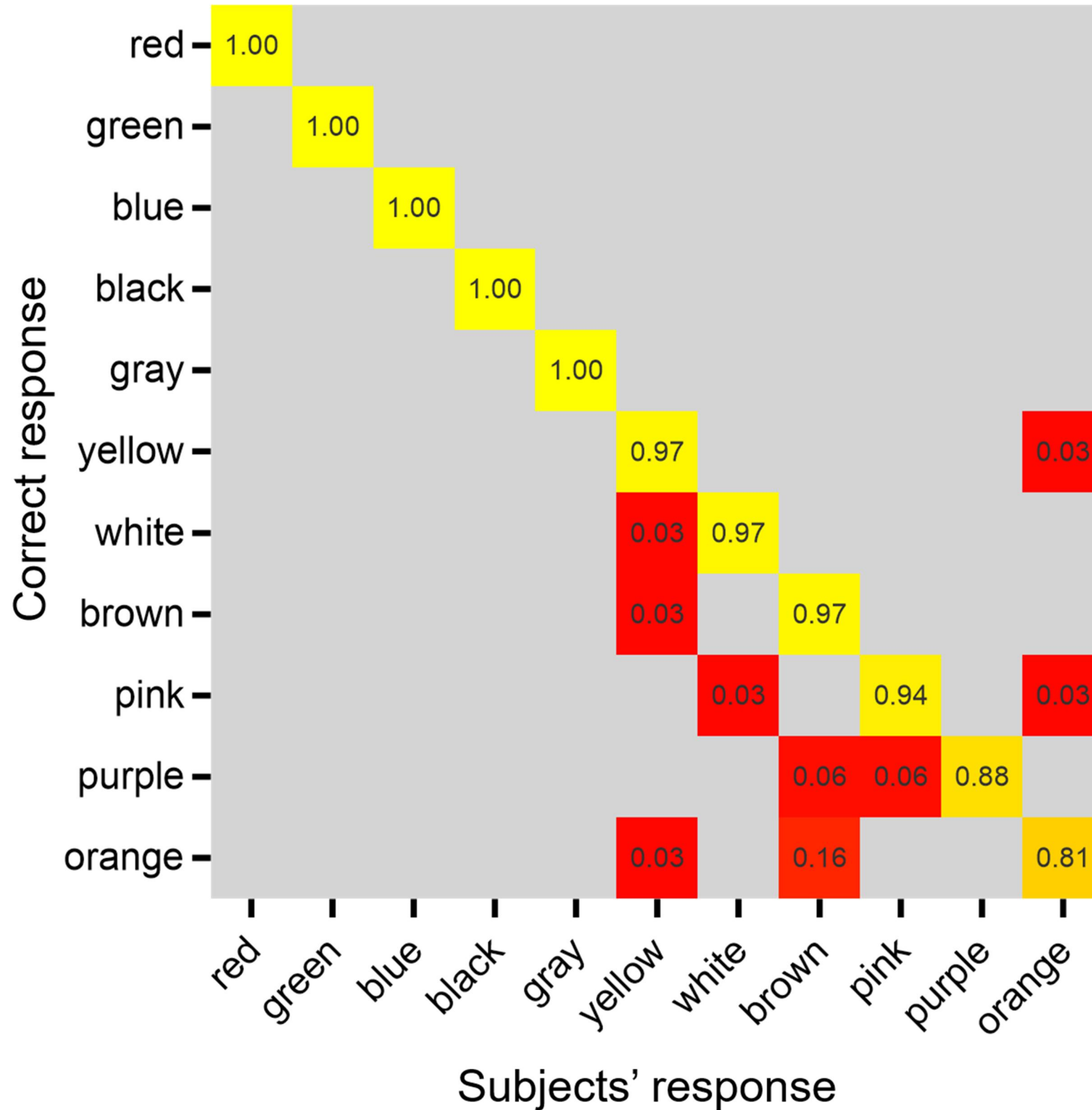

### Patients (n=32)

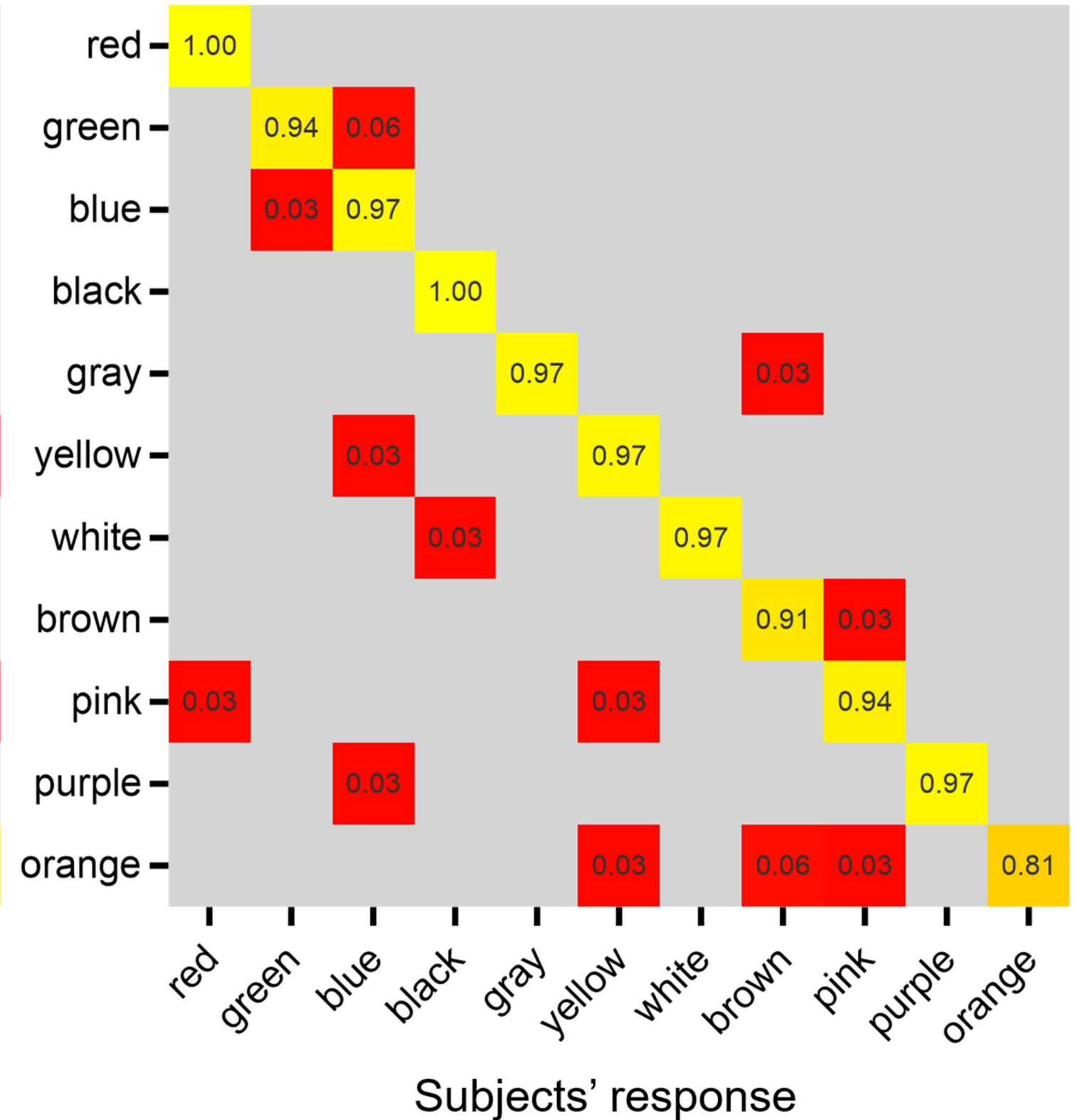

Supplement: S4 Fig — The overall proportion of “orange” and “brown” words in the patient group was less than 100% because two patients thought that the two color words did not match any color patches presented in the image. The data underlying this figure are available in S1 Data. (PDF) [file pbio.3003161.s004.pdf]

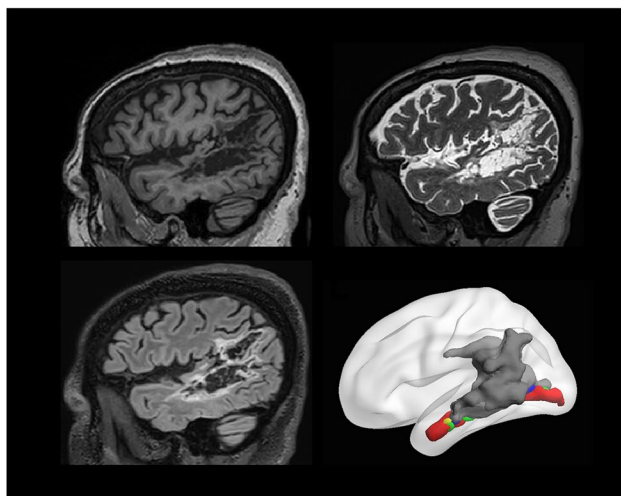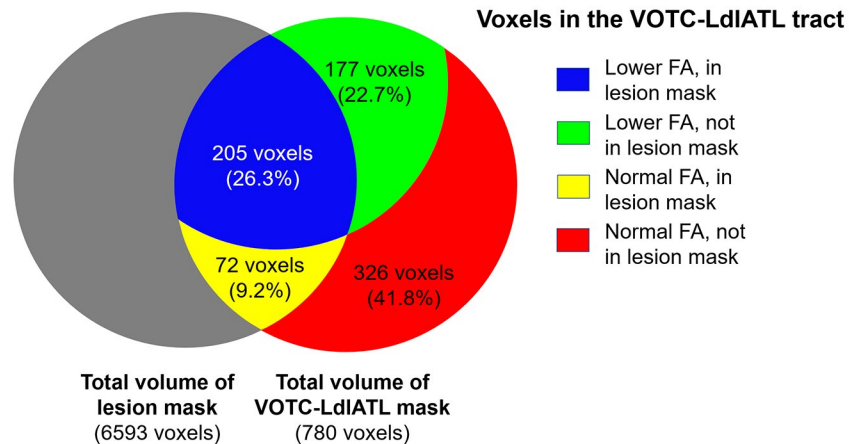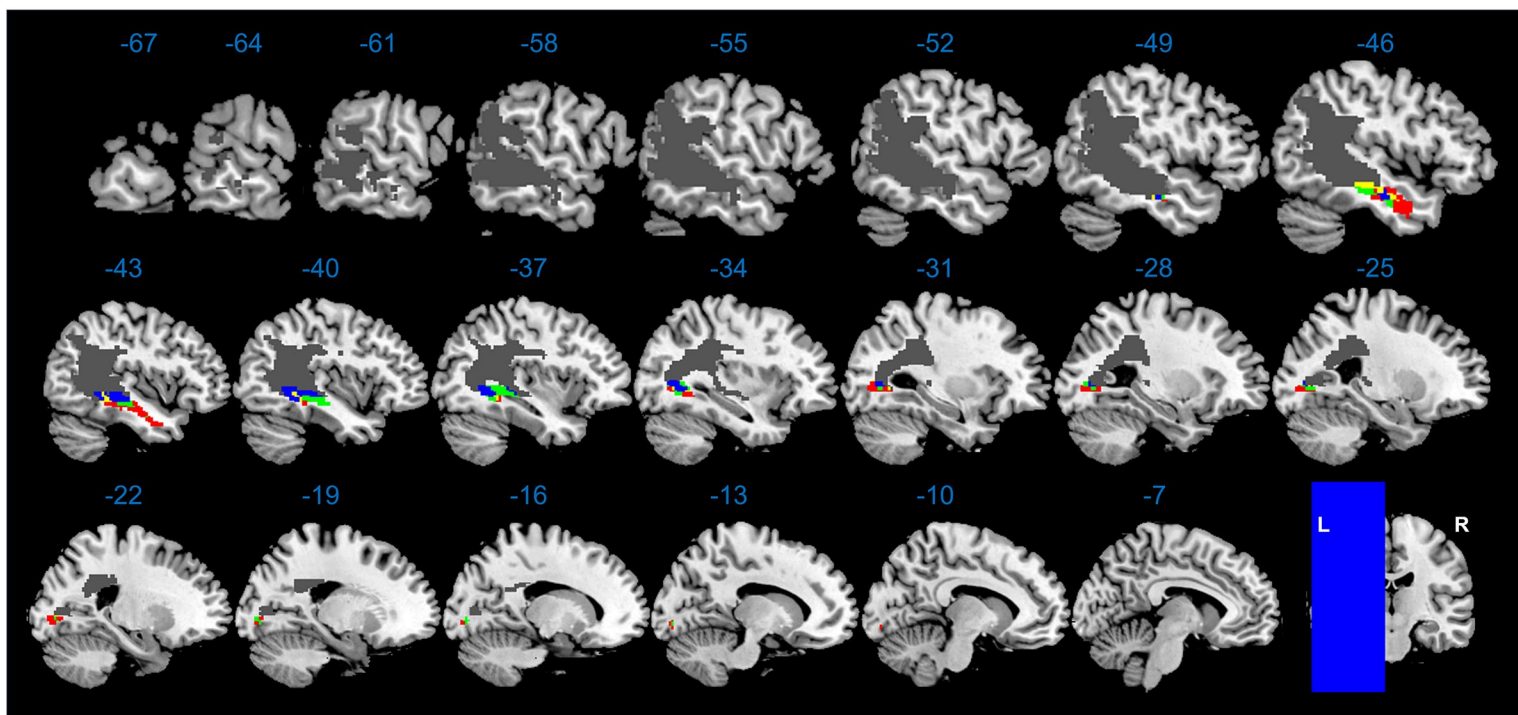

Supplement: S6 Fig — Lesion profile. The three sagittal slice images (in the top left panel) show T1-weighted, T2-weighted, and FLAIR T2-weighted images of the same slice in the native space. The cortical map and the slice images (in the bottom panel) show the overlap of the patient’s lesions with the VOTC-LdlATL tract in the MNI space, illustrating that the patient’s VOTC-LdlATL tract was severely lesioned. Two types of lesions were considered here: manually drawn lesion masks and voxels with significantly lower FA values. The low-FA voxels, defined as voxels with FA values significantly lower than those of healthy controls according to the single-case modified t test [76]; p < 0.05, one-tailed], was to quantify invisible lesions. The top right panel shows the extent of overlap in the proportion of lesions within the VOTC-LdlATL tract. The manually drawn lesion mask overlapped with 35.5% of the VOTC-LdlATL tract, consisting of 26.3% with significantly lower FA values (blue area) and 9.2% (yellow area) whose FA values were in the normal range. 22.7% of the tract (green area) was found to have lower FA values and was outside the manually drawn lesion mask. The red area shows the portion of the VOTC-LdlATL tract that was not affected in the patient; the gray area shows the lesion outside the VOTC-LdlATL tract. Brain imaging results were visualized using BrainNet Viewer (version 1.7; https://www.nitrc.org/projects/bnv/; RRID: SCR_009446), or MRIcron (version 1.0.20190902; https://www.nitrc.org/projects/mricron; RRID: SCR_002403). Abbreviations: VOTC, ventral occipitotemporal cortex; L, left; dlATL, dorsolateral anterior temporal lobe; FA, fractional anisotropy. (PDF) [file pbio.3003161.s006.pdf]
