# Supplementary material for: Object knowledge representation in the human visual cortex requires a connection with the language system
Source: PLoS Biol. 2025 May 20;23(5):e3003161. doi: 10.1371/journal.pbio.3003161 (PMC12091770; doi:10.1371/journal.pbio.3003161)
Supplement: S2 Fig — The red color scale (from 0 to 1) indicates the group-level probability of a voxel belonging to a white-matter connection. (A) Group-level probability maps of white-matter connectivity between the bilateral VOTC and the left dlATL (top) and right dlATL (bottom), respectively, at the individual threshold of 0.1 (the threshold used in the main analysis). (B) Group-level probability map between the bilateral VOTC and the left dlATL at an individual threshold of 0.001. Brain results were visualized using MRIcron (version 1.0.20190902; https://www.nitrc.org/projects/mricron; RRID: SCR_002403). Abbreviations: VOTC, ventral occipitotemporal cortex; dlATL, dorsolateral anterior temporal lobe. (PDF) [file pbio.3003161.s002.pdf]

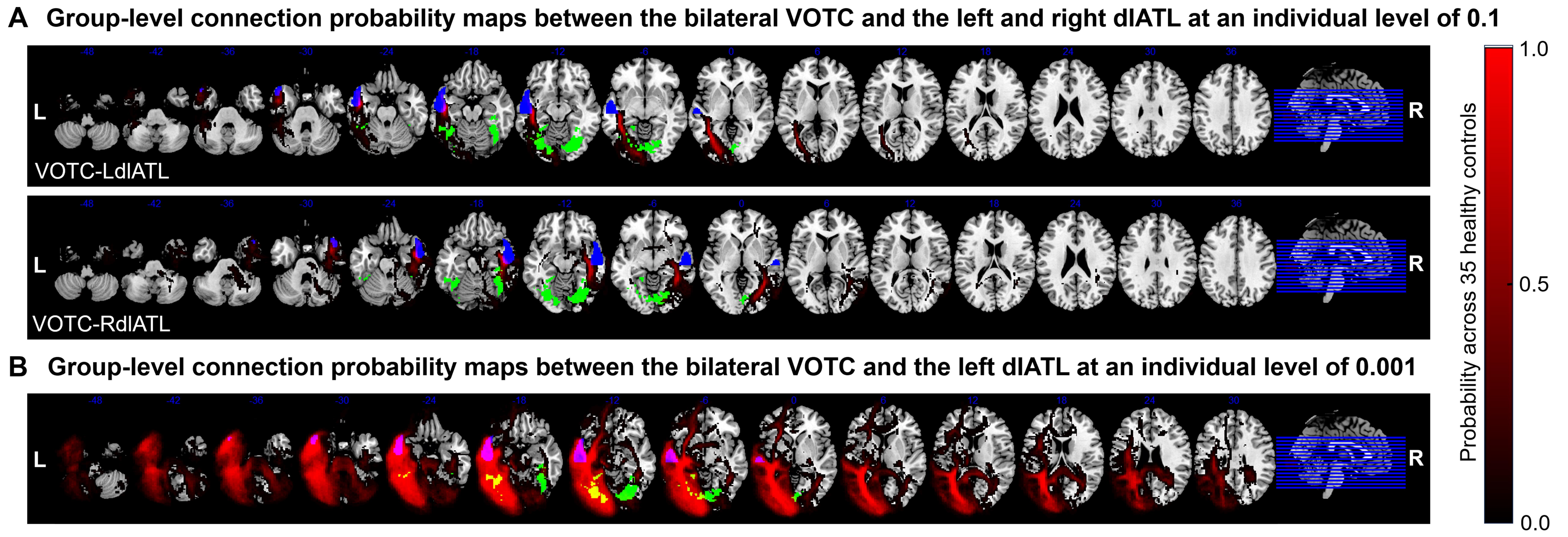
A Group-level connection probability maps between the bilateral VOTC and the left and right dlATL at an individual level of 0.1
L
R
VOTC-LdlATL
VOTC-RdlATL
B Group-level connection probability maps between the bilateral VOTC and the left dlATL at an individual level of 0.001
Probability across 35 healthy controls
1.0
0.5
0.0
