# Supplementary material for: Object knowledge representation in the human visual cortex requires a connection with the language system
Source: PLoS Biol. 2025 May 20;23(5):e3003161. doi: 10.1371/journal.pbio.3003161 (PMC12091770; doi:10.1371/journal.pbio.3003161)
Supplement: S3 Fig — The first row illustrates the seed regions for probabilistic tractography and the corresponding reconstructed white-matter tracts in healthy controls: VOTC-LpMTG, VOTC-LAG, VOTC-LIFGorb, VOTC-LIFG, and VOTC-LMFG. The second row shows the scatter plots between the mean FA values of these tracts and the VOTC object-color neural representation (Fisher-Z transformed r values). The third row shows the scatter plots between these tracts and object color behavior (the composite score across the verbal and non-verbal object color tasks). Similar to Fig 3, the behavioral outlier patient was removed from the scatter plots with object color behaviors and the reported partial rho and p-values were based on data from all the patients. The data underlying this figure are available in S1 Data. Brain imaging results were visualized using MRIcroGL (version 1.2.20210317; https://www.nitrc.org/projects/mricrogl). Abbreviations: VOTC, ventral occipitotemporal cortex; L, left; pMTG, posterior middle temporal gyrus; AG, angular gyrus; IFGorb, inferior frontal gyrus, orbital part; MFG, middle frontal gyrus; FA, fractional anisotropy. (PDF) [file pbio.3003161.s003.pdf]

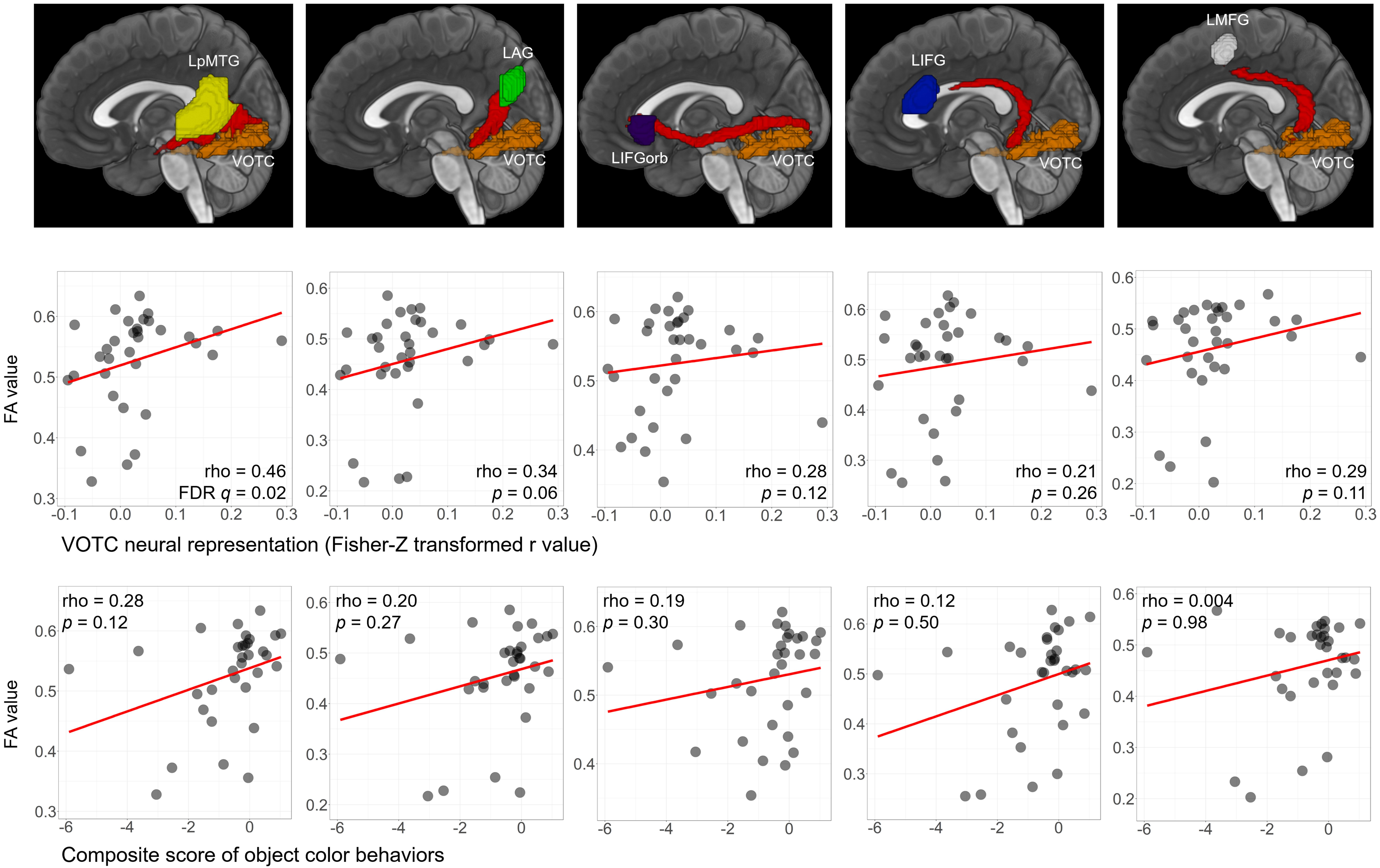
LpMTG
VOTC
LAG
VOTC
LIFGorb
VOTC
LIFG
VOTC
LMFG
VOTC
FA value
rho = 0.46
FDR q = 0.02
rho = 0.34
p = 0.06
rho = 0.28
p = 0.12
rho = 0.21
p = 0.26
rho = 0.29
p = 0.11
VOTC neural representation (Fisher-Z transformed r value)
FA value
rho = 0.28
p = 0.12
rho = 0.20
p = 0.27
rho = 0.19
p = 0.30
rho = 0.12
p = 0.50
rho = 0.004
p = 0.98
Composite score of object color behaviors
