# Supplementary material for: Object knowledge representation in the human visual cortex requires a connection with the language system
Source: PLoS Biol. 2025 May 20;23(5):e3003161. doi: 10.1371/journal.pbio.3003161 (PMC12091770; doi:10.1371/journal.pbio.3003161)
Supplement: S5 Fig — The six ATL subregions were taken from the HOA template (see Materials and methods). The first row displays the seed regions for probabilistic tractography and the corresponding reconstructed white-matter tracts: VOTC-LTP, VOTC-LASTG, VOTC-LAMTG, VOTC-LAITG, VOTC-LATFC, and VOTC-LAPG. The second row illustrates the correlations between these six tracts and VOTC object color neural representation. The third row shows the correlations between these six tracts and object color behavior (the composite score across the verbal and non-verbal object color tasks). The white-matter connections between the three ventral subregions (LAITG, LATFC, and LAPG) and the VOTC were disrupted and had fewer than 100 voxels under the thresholds used in the main analysis (individual-level threshold of 0.1 and a group-level threshold of 0.5, masked by the SPM12 white-matter map with probability > 0.4). The tracts shown here were obtained using more lenient thresholds (individual-level threshold of 0.1 and group-level threshold of 0.4, without the explicit white-matter mask). The correlations reported in the scatter plots were based on the tracts reconstructed using the more lenient thresholds, and similar correlations were observed with the tracts under the default thresholds. Similar to Fig 3, the behavioral outlier patient was removed from the scatter plots with object color behaviors and the reported partial rho and p-values were based on data from all the patients. The data underlying this figure are available in S1 Data. Brain imaging results were visualized using MRIcroGL (version 1.2.20210317; https://www.nitrc.org/projects/mricrogl). Abbreviations: VOTC, ventral occipitotemporal cortex; L, left; TP, temporal pole; ASTG, anterior superior temporal gyrus; AMTG, anterior middle temporal gyrus; AITG, anterior inferior temporal gyrus; ATFC, anterior temporal fusiform cortex; APG, anterior parahippocampal gyrus; FA, fractional anisotropy; HOA, Harvard-Oxford Atlas. (PDF) [file pbio.3003161.s005.pdf]

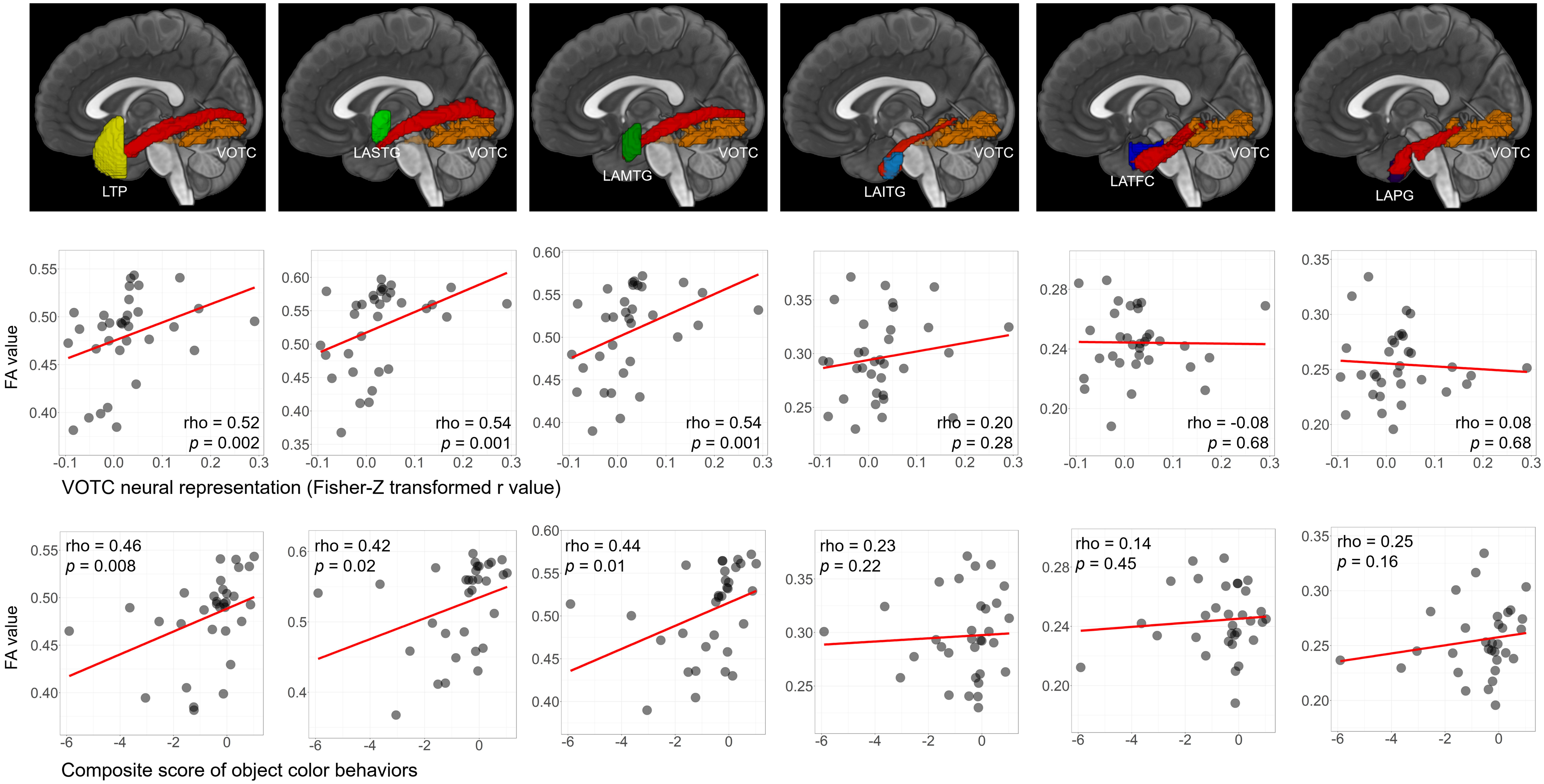

LTP
VOTC
LASTG
LAMTG
LAITG
LATFC
LAPG
rho = 0.52
p = 0.002
rho = 0.54
p = 0.001
rho = 0.54
p = 0.001
rho = 0.20
p = 0.28
rho = -0.08
p = 0.68
rho = 0.08
p = 0.68
FA value
VOTC neural representation (Fisher-Z transformed r value)
rho = 0.46
p = 0.008
rho = 0.42
p = 0.02
rho = 0.44
p = 0.01
rho = 0.23
p = 0.22
rho = 0.14
p = 0.45
rho = 0.25
p = 0.16
FA value
Composite score of object color behaviors
