# Supplementary material for: Object knowledge representation in the human visual cortex requires a connection with the language system
Source: PLoS Biol. 2025 May 20;23(5):e3003161. doi: 10.1371/journal.pbio.3003161 (PMC12091770; doi:10.1371/journal.pbio.3003161)
Supplement: S1 Table — (DOCX) [file pbio.3003161.s007.docx]

**S1 Table.** Background information of the 33 stroke patients.

| **ID** | **Age**  **(year)** | **Gender (M/F)** | **Education (year)** | **MMSE (score)** | **Post-onset of disease**  **(month)** | **Lesion volume**  **(mm^3^)** | **Lesion site** | | | | | **Notes** |
| --- | --- | --- | --- | --- | --- | --- | --- | --- | --- | --- | --- | --- |
|  |  |  |  |  |  |  | **Left hemisphere** | | **Right hemisphere** | | **Brain stem** |  |
|  |  |  |  |  |  |  | **Gray-matter** | **White-matter** | **Gray-matter** | **White-matter** |  |  |
| 1 | 44 | M | 14 | 12 | 13 | 112330 | F, P, T, Ins, BG, Thal, Amy | * | - | - | - |  |
| 2 | 40 | M | 12 | 23 | 11 | 140122 | F, P, BG | * | F, P, O, T, BG | * | - |  |
| 3 | 33 | M | 16 | 26 | 156 | 89461 | F, P, T, Ins, BG | * | F, P, T, Ins | * | - | Potential old lesions |
| 4 | 42 | M | 15 | 29 | 32 | 2377 | F, P, O, BG | * | - | - | - |  |
| 5 | 54 | M | 14 | 28 | 26 | 236545 | - | - | F, P, O, T, Lim, Ins, BG, Thal, Amy | * | - |  |
| 6 | 53 | F | 5 | 18 | 10 | 46147 | - | - | F, P, Ins, BG, Thal | * | - |  |
| 7 | 39 | M | 16 | 30 | 6 | 14536 | - | - | F, T | * | - | Potential old lesions |
| 8 | 65 | F | 9 | 29 | 33 | 3761 | O | - | F, Ins | * | - |  |
| 9 | 57 | M | 8 | 28 | 33 | 2800 | O | * | - | - | - |  |
| 10 | 57 | M | 10 | 28 | 252 | 35169 | F, T, Ins, BG, Thal | * | - | - | - |  |
| 11 | 63 | F | 14 | 14 | 22 | 52675 | P, T, O, Ins, Thal | * | - | - | - |  |
| 12 | 47 | M | 16 | 21 | 12 | 28091 | F, P, T, Ins, BG, Thal | * | - | - | - |  |
| 14 | 65 | F | 10 | 28 | 21 | 3359 | BG | * | - | - | - |  |
| 16 | 51 | F | 8 | 28 | 4 | 172987 | - | - | F, P, T, O, Ins, BG | * | - |  |
| 17 | 57 | M | 8 | 29 | 16 | 61591 | - | - | P, T, O | * | - |  |
| 18 | 63 | F | 11 | 23 | 180 | 12488 | F, P, O | * | - | - | - | Premorbid distant visual problem (not improved with correction) |
| 19 | 59 | M | 8 | 30 | 11 | 4717 | - | - | P, O | * | - |  |
| 21 | 56 | M | 8 | 23 | 7 | 20989 | F, P, T, O, BG | * | - | - | - |  |
| 22 | 49 | F | 15 | 30 | 7 | 1016 | - | - | F, P, Ins | - | - |  |
| 23 | 51 | M | 11 | 29 | 5 | 3322 | BG, Thal | * | - | - | - |  |
| 24 | 62 | F | 13 | 25 | 3 | 1358 | BG | * | - | - | - |  |
| 25 | 60 | M | 13 | 27 | 11 | 22168 | P, O, T | * | - | - | - |  |
| 26 | 30 | M | 16 | 27 | 10 | 94298 | F, P, T, Ins, BG | * | - | - | - |  |
| 27 | 53 | M | 9 | 19 | 5 | 74683 | F, P, Ins, BG, Thal | * | - | - | * |  |
| 28 | 34 | M | 10 | 28 | 4 | 37687 | T, O | * | CB | - | - |  |
| 29 | 53 | M | 8 | 28 | 27 | 50328 | F, P, Ins, BG, Thal | * | - | - | - |  |
| 30 | 44 | M | 12 | 28 | 29 | 22871 | F, P, T, BG, Thal | * | - | - | - |  |
| 31 | 57 | M | 8 | 27 | 3 | 4609 | - | - | F, P, O | - | - |  |
| 32 | 59 | M | 12 | 25 | 17 | 5887 | - | - | Ins, Thal | * | - |  |
| 33 | 54 | M | 5 | 28 | 10 | 53155 | - | - | P, O, T | * | - |  |
| 34 | 53 | M | 12 | 29 | 8 | 68496 | - | - | F, P, O, T, Ins, BG, Thal | * | - |  |
| 35 | 33 | M | 15 | 30 | 6 | 852 | F, P, T | * | - | - | - |  |
| 36 | 64 | M | 8 | 27 | 3 | 2524 | F, P | * | - | - | - |  |

*Abbreviations: MMSE, Mini-Mental State Examination; M, male; F, female; F, frontal lobe; P, parietal lobe; O, occipital lobe; T, temporal lobe; Lim, limbic lobe; Ins, insula; BG, basal ganglia; Thal, thalamus; Amy, Amygdala; CB, cerebellum; *, lesioned; -, Intact.*
