# Supplementary material for: Object knowledge representation in the human visual cortex requires a connection with the language system
Source: PLoS Biol. 2025 May 20;23(5):e3003161. doi: 10.1371/journal.pbio.3003161 (PMC12091770; doi:10.1371/journal.pbio.3003161)
Supplement: S2 Table — (DOCX) [file pbio.3003161.s008.docx]

**S2 Table.** Raw accuracy and standardized scores of each stroke patient in the four neuropsychological tests.

| **ID** | **Object color knowledge behavior** | | | | |  | **Control behavior** | | | | |
| --- | --- | --- | --- | --- | --- | --- | --- | --- | --- | --- | --- |
|  | **Grayscale picture-color word matching task (verbal, 60 trials)** | |  | **Object color true/false judgment task (non-verbal, 60 trials)** | |  | **Word-picture matching task**  **(40 trials)** | |  | **Color patch matching task**  **(11 trials)** | |
|  | **Accuracy** | **Standardized score** |  | **Accuracy** | **Standardized score** |  | **Accuracy** | **Standardized score** |  | **Accuracy** | **Standardized score** |
| 1 | 0.83 | -2.53* |  | 0.97 | 0.05 |  | 0.98 | -0.69 |  | 1.00 | 0.80 |
| 2 | 0.78 | -3.49*** |  | 0.72 | -8.32*** |  | 0.80 | -10.29*** |  | 1.00 | 1.13 |
| 3 | 0.88 | -1.61 |  | 0.97 | -0.10 |  | 1.00 | 0.31 |  | 0.91 | -0.55 |
| 4 | 0.97 | 0.64 |  | 0.95 | -0.64 |  | 1.00 | 0.62 |  | 1.00 | 0.75 |
| 5 | 0.92 | -0.42 |  | 0.97 | -0.03 |  | 1.00 | 0.68 |  | 1.00 | 0.50 |
| 6 | 0.52 | -9.64*** |  | 0.63 | -11.27*** |  | 0.85 | -7.49*** |  | 0.82 | -1.83 |
| 7 | 0.97 | 0.49 |  | 0.98 | 0.40 |  | 0.95 | -2.27* |  | 1.00 | 0.74 |
| 8 | 0.97 | 0.95 |  | 0.93 | -1.48 |  | 1.00 | 0.50 |  | 1.00 | 0.31 |
| 9 | 0.88 | -0.40 |  | 0.87 | -2.79** |  | 0.95 | -1.56 |  | 0.82 | -1.87 |
| 10 | 0.88 | -0.69 |  | 0.97 | 0.43 |  | 0.95 | -1.75 |  | 0.91 | -0.63 |
| 11 | 0.82 | -3.48** |  | 0.92 | -2.62* |  | 0.95 | -2.76** |  | 0.64 | -5.99*** |
| 12 | 0.83 | -2.80** |  | 0.97 | -0.22 |  | 0.95 | -2.29* |  | 0.64 | -5.34*** |
| 14 | 0.95 | 0.39 |  | 1.00 | 0.72 |  | 1.00 | 0.41 |  | 1.00 | 0.21 |
| 16 | 0.88 | -1.03 |  | 0.97 | -0.07 |  | 1.00 | 0.63 |  | 1.00 | 0.83 |
| 17 | 0.90 | 0.02 |  | 0.97 | 0.67 |  | 0.98 | -0.16 |  | 1.00 | 1.04 |
| 18 | 0.85 | -2.23* |  | 0.90 | -2.84** |  | 0.95 | -2.48* |  | / | / |
| 19 | 0.93 | 0.84 |  | 0.98 | 1.21 |  | 1.00 | 1.23 |  | 1.00 | 0.98 |
| 21 | 0.92 | 0.44 |  | 0.95 | 0.09 |  | 1.00 | 1.24 |  | 1.00 | 1.07 |
| 22 | 0.97 | 0.05 |  | 0.98 | -0.34 |  | 1.00 | -0.02 |  | 1.00 | 0.16 |
| 23 | 0.92 | -0.01 |  | 0.95 | -0.23 |  | 1.00 | 0.97 |  | 1.00 | 0.91 |
| 24 | 0.92 | -0.87 |  | 1.00 | 0.38 |  | 1.00 | 0.14 |  | 1.00 | -0.02 |
| 25 | 0.90 | -0.69 |  | 0.98 | 0.59 |  | 0.98 | -0.64 |  | 1.00 | 0.43 |
| 26 | 0.92 | -0.78 |  | 1.00 | 1.06 |  | 1.00 | 0.55 |  | 0.91 | -0.46 |
| 27 | 0.83 | -1.79* |  | 1.00 | 1.72 |  | 0.93 | -3.04** |  | 0.82 | -1.86 |
| 28 | 0.97 | 1.33 |  | 0.88 | -2.29* |  | 0.93 | -3.09** |  | 1.00 | 1.52 |
| 29 | 0.92 | 0.43 |  | 0.98 | 1.25 |  | 1.00 | 1.25 |  | 1.00 | 1.16 |
| 30 | 0.87 | -1.40 |  | 0.90 | -2.03* |  | 0.95 | -1.90* |  | 0.91 | -0.45 |
| 31 | 0.87 | -0.80 |  | 0.97 | 0.67 |  | 1.00 | 1.24 |  | 1.00 | 1.04 |
| 32 | 0.93 | 0.27 |  | 0.88 | -2.74** |  | 0.98 | -0.54 |  | 1.00 | 0.56 |
| 33 | 0.73 | -3.69*** |  | 0.83 | -3.58** |  | 0.95 | -1.27 |  | 1.00 | 1.45 |
| 34 | 0.88 | -0.98 |  | 0.97 | 0.22 |  | 0.95 | -1.93* |  | 1.00 | 0.74 |
| 35 | 0.97 | 0.61 |  | 1.00 | 1.16 |  | 1.00 | 0.64 |  | 1.00 | 1.02 |
| 36 | 0.87 | -0.78 |  | 0.95 | 0.02 |  | 0.98 | -0.18 |  | 1.00 | 0.84 |

Patient 018 did not participate in the color patch matching task, and her score for this task is marked with a “/”. Significance: *p < 0.05, **p < 0.01, ***p < 0.001 (one-tailed test, based on the Bayesian test for a Deficit allowing for Covariates (Crawford et al., 2011)).
