# Supplementary material for: Object knowledge representation in the human visual cortex requires a connection with the language system
Source: PLoS Biol. 2025 May 20;23(5):e3003161. doi: 10.1371/journal.pbio.3003161 (PMC12091770; doi:10.1371/journal.pbio.3003161)
Supplement: S3 Table — (DOCX) [file pbio.3003161.s009.docx]

**S3 Table.** The anatomical properties of the white-matter tracts connecting the VOTC and the left language regions in healthy controls.

|  | **Tract** | **Tract size (mm^3^)** |  | **Overlay percentage on the Johns Hopkins University template tract** |
| --- | --- | --- | --- | --- |
| 1 | VOTC-LdlATL | 6240 |  | Forceps_major: 2.9%, IFOF_L: 24.1%, ILF_L: 50.6% |
| 2 | VOTC-LpMTG | 5248 |  | Forceps_major: 0.2%, SLF_L: 4.1%, IFOF_L: 12.5%, ILF_L: 31.2%, SLF_temporal_part_L: 71.4% |
| 3 | VOTC-LAG | 2200 |  | IFOF_L: 0.1%, SLF_L: 1.0%, ILF_L: 2.7%, SLF_temporal_part_L: 42.9% |
| 4 | VOTC-LIFGorb | 4144 |  | SLF_L: 0.2%, Forceps_major: 2.0%, ILF_L: 15.3%, UF_L: 23.2%, IFOF_L: 30.3% |
| 5 | VOTC-LIFG | 2368 |  | IFOF_L: 0.7%, ILF_L: 1.6%, SLF_L: 15.4%, SLF_temporal_part_L: 71.4% |
| 6 | VOTC-LMFG | 2400 |  | IFOF_L: 0.3%, ILF_L: 3.1%, SLF_L: 7.8%, SLF_temporal_part_L: 42.9% |

*Abbreviations: VOTC, ventral occipital temporal cortex; L, left; dlATL, dorsolateral anterior temporal lobe; pMTG, posterior middle temporal gyrus; AG, angular gyrus; IFGorb, inferior frontal gyrus, orbital part; MFG, middle frontal gyrus; IFOF,* *inferior fronto-occipital fasciculus; ILF, inferior longitudinal fasciculus; SLF, superior longitudinal fasciculus; UF,* *uncinate fasciculus.*
