# Supplementary material for: Object knowledge representation in the human visual cortex requires a connection with the language system
Source: PLoS Biol. 2025 May 20;23(5):e3003161. doi: 10.1371/journal.pbio.3003161 (PMC12091770; doi:10.1371/journal.pbio.3003161)
Supplement: S4 Table — The data underlying this table are available in S1 Data. (DOCX) [file pbio.3003161.s010.docx]

**S4 Table.** Validation of the VOTC object color neural representation effects (correlations with the VOTC-LdlATL white-matter connection and object color behaviors), using VOTC-color-knowledge masks with different individual-level and group-level thresholds.

| **VOTC-color-knowledge mask** | | | **VOTC-LdlATL white-matter connection**  **(partial rho)** | **Object color behavior (partial rho)** | | |
| --- | --- | --- | --- | --- | --- | --- |
| **Individual level**  **(top n voxels)** | **Group level**  **(probability across healthy controls)** | **Mask size**  **(voxel)** |  | **Composite score** | **Verbal color** | **Non-verbal color** |
| 300 | 0.15 | 1025 | 0.34^#^ | -0.09 | -0.24 | -0.05 |
| 300 | 0.20 | 391 | 0.43* | -0.02 | -0.23 | 0.02 |
| **300** | **0.25** | **105** | **0.56***** | **0.11** | **-0.11** | **0.14** |
| 300 | 0.30 | 61 | 0.47** | 0.08 | -0.12 | 0.12 |
| 300 | 0.35 | 26 | 0.50** | 0.07 | -0.10 | 0.11 |
| 200 | 0.25 | 22 | 0.47** | 0.05 | -0.11 | 0.10 |
| 250 | 0.25 | 57 | 0.47** | 0.07 | -0.13 | 0.12 |
| 350 | 0.25 | 217 | 0.48** | 0.05 | -0.17 | 0.08 |
| 400 | 0.25 | 341 | 0.44* | -0.01 | -0.22 | 0.03 |
| 450 | 0.25 | 565 | 0.38* | -0.04 | -0.22 | 0.003 |
| 500 | 0.25 | 689 | 0.36* | -0.04 | -0.24 | 0.01 |

The result of main analyses is in bold. Partial correlation coefficients are reported, controlling for total lesion volume. Significance: ^#^*p* < 0.1, **p* < 0.05, ***p* < 0.01, ****p* < 0.001 (two-tailed test). *Abbreviations:* *VOTC, ventral occipital temporal cortex; L, left; dlATL, dorsolateral anterior temporal lobe.*
