# Supplementary material for: Object knowledge representation in the human visual cortex requires a connection with the language system
Source: PLoS Biol. 2025 May 20;23(5):e3003161. doi: 10.1371/journal.pbio.3003161 (PMC12091770; doi:10.1371/journal.pbio.3003161)
Supplement: S5 Table — The data underlying this table are available in S1 Data. (DOCX) [file pbio.3003161.s011.docx]

**S5 Table.** Validation of the effects of the VOTC-LdlATL white-matter connection (with different individual-level and group-level thresholds, with or without the explicit white-matter mask) on VOTC object color neural representation and object color behaviors.

| **VOTC-LdlATL white-matter connection mask** | | | | **VOTC neural representation**  **(partial rho)** | **Object color behavior (partial rho)** | | |
| --- | --- | --- | --- | --- | --- | --- | --- |
| **Individual level**  **(****connectivity probability)** | **Group level**  **(probability across healthy controls)** | **White-matter mask**  **(Y/N)** | **Mask size**  **(voxel)** |  | **Composite score** | **Verbal color** | **Non-verbal color** |
| 0.05 | 0.50 | Y | 1409 | 0.54** | 0.48** | 0.42* | 0.33^#^ |
| **0.10** | **0.50** | **Y** | **780** | **0.56***** | **0.46**** | **0.41*** | **0.30^#^** |
| 0.15 | 0.50 | Y | 477 | 0.56*** | 0.41* | 0.37* | 0.26 |
| 0.20 | 0.50 | Y | 305 | 0.49** | 0.43* | 0.37* | 0.24 |
| 0.10 | 0.35 | Y | 1180 | 0.55** | 0.47** | 0.42* | 0.31^#^ |
| 0.10 | 0.40 | Y | 1081 | 0.55** | 0.46** | 0.40* | 0.30^#^ |
| 0.10 | 0.45 | Y | 927 | 0.55** | 0.45** | 0.40* | 0.29 |
| 0.10 | 0.55 | Y | 665 | 0.56*** | 0.45* | 0.41* | 0.29 |
| 0.10 | 0.60 | Y | 596 | 0.58*** | 0.45** | 0.40* | 0.30 |
| 0.10 | 0.50 | N | 781 | 0.56*** | 0.46** | 0.41* | 0.30^#^ |

The result of main analyses is in bold. Partial correlation coefficients are reported, controlling for total lesion volume. Significance: ^#^*p* < 0.1, **p* < 0.05, ***p* < 0.01, ****p* < 0.001 (two-tailed test). *Abbreviations: VOTC, ventral occipital temporal cortex; L, left; dlATL, dorsolateral anterior temporal lobe; white-matter mask, an explicit white-matter mask produced by binarizing the SPM12 white-matter probability map with a threshold of 0.4; Y, with the explicit white-matter mask; N, without the explicit white-matter mask.*
