# Supplementary material for: Object knowledge representation in the human visual cortex requires a connection with the language system
Source: PLoS Biol. 2025 May 20;23(5):e3003161. doi: 10.1371/journal.pbio.3003161 (PMC12091770; doi:10.1371/journal.pbio.3003161)
Supplement: S6 Table — The data underlying this table are available in S1 Data. (DOCX) [file pbio.3003161.s012.docx]

**S6 Table.** Partial correlation coefficients between the VOTC-LdlATL tract integrity (mean FA value) and performances on the grayscale picture-color word matching (verbal) and object color true/false judgment (non-verbal) tasks, along with between-task comparisons of the correlations using Hotelling’s t-test, controlling for potential confounding factors.

| Control variables | | Verbal color  (partial rho) | Non-verbal color  (partial rho) | Verbal vs. non-verbal (Hotelling’s t value) |
| --- | --- | --- | --- | --- |
| *Broader effects of lesion* | | | | |
|  | TLV | 0.41* | 0.30^#^ | 0.61 |
|  | Seed GM damage & TLV | 0.51** | 0.42* | 0.58 |
|  | Visual perception pathway & TLV | 0.38* | 0.23 | 0.78 |
| *Related cognitive process* | | | | |
|  | Word-picture matching score & TLV | 0.29 | 0.15 | 0.64 |
|  | Color patch matching score & TLV | 0.26 | 0.26 | 0.00 |
| *Patient etiology* | | | | |
|  | Post-onset time & TLV | 0.44* | 0.25 | 1.08 |
|  | Excluding 2 patients with potential old lesions: TLV | 0.39* | 0.29 | 0.52 |

Significance: ^#^*p* < 0.1, **p* < 0.05, ***p* < 0.01 (two-tailed test). *Abbreviations: FA, fractional anisotropy; L, left; dlATL, dorsolateral anterior temporal lobe; TLV: total lesion volume;* *GM, gray-matter.*
