# Supplementary material for: Object knowledge representation in the human visual cortex requires a connection with the language system
Source: PLoS Biol. 2025 May 20;23(5):e3003161. doi: 10.1371/journal.pbio.3003161 (PMC12091770; doi:10.1371/journal.pbio.3003161)
Supplement: S1 Text — (DOCX) [file pbio.3003161.s014.docx]

**S1 Text. A case of damage to the VOTC-LdlATL tract.**

The main analyses revealed that across patients, the integrity of the WM connection between LdlATL and VOTC matters for the strength of color knowledge neural representation in the VOTC and for object color knowledge behavior. Here we present an in-depth case profile, focusing on a patient with damage to the VOTC-LdlATL tract, associated with the group observations, for illustrative purposes (**S6 Fig**).

Patient 011 was a 63-year-old, right-handed female with 14 years of formal education. Prior to her illness, she had retired from her job in accounting. She suffered an ischemic stroke in the territory of the left middle cerebral artery in February 2020. She participated in our study 22 months after her stroke. Her brain lesions (manually drawn) were in the left parietal, temporal, and occipital lobes, as well as the insula and thalamus. The VOTC-LdlATL tract was severely lesioned, with 58.2% of the voxels of the tract located in the lesion mask and/or exhibiting significantly lower FA values (**S6 Fig**). The voxels in the VOTC-color-perception mask were not affected.

At the time of testing, the patient was fully conscious, with no hemiplegia. Her speech was largely fluent and she complained of slight problems with auditory comprehension. Her MMSE score was 14. She had normal color vision on the color-plate test and was eager to participate in our study. She performed well in the task fMRI scanning (91.1%, 102 out of 112 trials). Her RSA effects of object color representation in the VOTC were negative (Fisher-Z-transformer r value: -0.05) and below the average of healthy controls (0.02 ± 0.05 (SD)). Her performance (accuracy) in the verbal object color task was 81.7% (49/60), and her performance in the non-verbal object color task was 91.7% (55/60), both of which indicated impairment compared with the performance of the healthy controls (verbal color task: effect size = − 3.48, *p* = 0.002; non-verbal color task: effect size = − 2.62, *p* = 0.01). For example, she incorrectly responded “no” to the pairing of the word “yellow” and a grayscale picture of a pineapple, and responded “yes” to a pineapple colored red.
