# Supplementary material for: Object knowledge representation in the human visual cortex requires a connection with the language system
Source: PLoS Biol. 2025 May 20;23(5):e3003161. doi: 10.1371/journal.pbio.3003161 (PMC12091770; doi:10.1371/journal.pbio.3003161)
Supplement: Attachment — Submitted filename: Response to reviewers.docx [file pbio.3003161.s018.docx]

We greatly appreciate the constructive comments and suggestions from the Reviewers. We have carefully revised the manuscript to address the Reviewers’ concerns by performing additional analyses and making substantial clarifications. The manuscript has been much improved. Thank you. Below we explain how each comment was addressed.

REVIEWS:

Reviewer #1: Summary. This study examined the necessary role of language regions in the neural representation of semantic information, specifically for knowledge of object color. Thus, the research addressed fundamental questions about the extent to which semantic knowledge representations are sensory in nature or have language-based aspects.  Using representational similarity analyses (RSA) of fMRI data from healthy individuals, regions of the ventral-occipital cortex with the greatest involvement in the representation of object color were identified and used to create a color-knowledge mask. Probabilistic tractography of diffusion imaging was successful in identifying white matter tracts connecting this color-knowledge mask to six language regions identified using a language localizer task contrasting the processing of sentences vs. lists of nonwords.
33 patients with lesions from stroke that spared posterior regions were studied with the same behavioral and imaging protocols as for the healthy subjects. For the patients, the tract with the highest correlation between FA values and the mean RSA values in the VOTC mask had a termination in the left dorsal-lateral anterior temporal lobe (LdlATL), a region that had been implicated in color knowledge in the congenitally blind. The results suggest that input from the language region determines how well object color knowledge is represented in the VOTC. Also, behavioral scores on the color tasks were significantly correlated with the integrity of the tract linking VOTC to LdlATL but not significantly correlated with any other tract.  Interestingly the behavioral scores were not related to RSA decoding in the VOTC.

Critique. The results strongly implicate a role for language representations in semantic knowledge of object color. The research was carefully done, ruling out alternative explanations for the significant relations involving the LdlATL, such as damage to the gray matter regions at the termini. However, there are several issues that should be addressed before the paper is considered for publication.

Response: Thank you for the positive evaluation of the study. We have carefully revised the paper to address your suggestions below.

Q1: 1) More information is needed about the tractography. The identified language regions were all in the left hemisphere but the VOTC included bilateral regions. How are tracts identified that go from the right hemisphere portions of VOTC to the left hemisphere language regions? Presumably the tracts would have to include the corpus callosum or some other tract connecting the two hemispheres. Or perhaps only left hemisphere parts of the VOTC mask were used, which is suggested by the presentation of the analysis of homologous right hemisphere tracts in lines 338-340.

Response: Thank you for this question. Indeed, while the bilateral VOTC was used as a seed region in the tractography analysis, the observed tracts ran mainly from the left VOTC to the left language regions. The white matter mask obtained using the current threshold contained no tracts from the right VOTC to the left language regions: individual level, 0.1 (connectivity probability); group level, 0.5 (connections exist in at least 50% of healthy controls); in the explicit WM mask (probability > 0.4 in the WM probability map in SPM12). In the revised manuscript, we added a Supplementary Figure (**S2 Fig**) showing the group-level connection probability maps with no group-level threshold between the bilateral VOTC and the left and right dlATL, respectively. Within-hemispheric connections between the VOTC and dlATL (in both the left and right hemispheres) were predominant. As the reviewer predicted, when we relaxed the individual-level threshold to 0.001, the connection probability map included connections from the right VOTC to the left dlATL through the corpus callosum (**S2B Fig**). This clarification has been added to the revised manuscript: “Note that while we used a bilateral VOTC color mask, under current thresholds for obtaining the WM tracts, no tracts connecting the right VOTC mask and the left language regions were included. Those tracts connecting the right VOTC mask and the left language regions through the corpus callosum could be observed when the individual level threshold was loosened to 0.001 (see **S2 Fig** for a full WM connection probabilistic map between the bilateral VOTC and the dlATL, for example)” (Results section, page 5).

In light of this comment, we also examined whether connection-with-left-dlATL integrity affects VOTC color knowledge representation, mainly in the left hemisphere. The results showed that when analyzed separately, both the left and right VOTC color knowledge representations were significantly affected by the integrity of the VOTC-LdlATL tract. The effect on the right VOTC might arise from strong functional connectivity between homologous regions that is supported by indirect or direct WM connections via the corpus callosum (not analyzed here given the subthreshold tractography results). We added this new analysis to the revised manuscript: “When the VOTC in the two hemispheres were analyzed separately, both neural representations were significantly correlated with the integrity of the VOTC-LdlATL tract (left VOTC: partial rho = 0.38, *p* = 0.03; right VOTC: partial rho = 0.51, *p* = 0.003)” (Results section, page 6).

Q2: 2) What is to be made of the fact that there was no correlation between patient behavior and the RSA values for the VOTC? Given that the strength of the WM tracts relates to decoding of color in the VOTC, implying an influence of language on color knowledge, why wouldn't that degree of color knowledge relate to behavior?

Response: We very much appreciate this question. Indeed, we were also initially puzzled by this. We speculate that the color knowledge representation in the VOTC contributes only partly to behavior (verbal or non-verbal) that is not strong/unique enough to be seen. A more trivial possible reason is that we selected patients with lesions outside the VOTC (given the primary interest in the connection) and the cohort is thus not optimal for revealing the effects of the VOTC. Alternatively, behavioral performance is the readout of many types of representations. While the VOTC neural activity codes color knowledge in ways that are 1) sensory-derived (overlapping with color perception, which is absent in congenitally blind individuals), and 2) modulated by language connection (results here), the behavioral performance for a typical object color knowledge task may (also) pull from other types of representations stored elsewhere, e.g., the integration of such VOTC representations and those in the LdlATL. Whether the effects of VOTC are visible in behavioral tasks that rely more directly on sensory-derived representations remains to be explored.

We have discussed these considerations in the revised manuscript: “Note that while the VOTC functional integrity was significantly modulated by its connection with the LdlATL, its correlation with object color behavior was not significant. This null effect should be interpreted with caution. As we deliberately selected stroke patients whose lesions largely spared the VOTC, this patient cohort may not be optimal for revealing the effects of the VOTC. Another possibility is that behavior is the readout of many types of representations, and the performance for judging object color knowledge tested here may (also) pull from other types of representations stored elsewhere, i.e., the integration of such VOTC representations and those in the LdlATL. The key point is that, in explaining behavior, the effects of the VOTC-LdlATL connection are beyond the potential effects of VOTC functionality.” (Discussion section, page 10).

Q3: 3) While the authors point out that the language processing involved was minimal in the tasks with a verbal component, greater emphasis should be placed on the fact that the significant correlations observed for the stroke patients persisted when only looking at the semantic tasks that were purely nonverbal.

Response: Thank you for this important observation. There was no significant difference between the effects of the two task modalities. We have now made this explicit in the revised manuscript, and provided results for the two tasks separately in the Supplementary materials: “The VOTC-LdlATL tract integrity was significantly correlated with the verbal object color behavior (rho = 0.41, *p* = 0.02) and marginally correlated with non-verbal object color behavior (partial rho = 0.30, *p* = 0.099), with no significant difference between these two tasks (Hotelling’s t values = 0.61, *p* = 0.55; **S6** **Table**).” (Results section, page 7).

Specific points.
Q4: Abstract. Something is said about possible implications of the findings for rehabilitation, but nothing is said in the paper about this topic.

Response: Thank you. In light of this comment, in the revised manuscript, we added the following explicit statement: “These findings, by highlighting the role of perception-language system connections in knowledge loss, motivate cognitive rehabilitation and/or brain stimulation treatment strategies that target such components.” (Discussion section, page 11).

Q5: Lines 139-155 This description was somewhat confusing. A color perception region was established and then apparently object color knowledge regions were only identified within that region. Is that known to be the case? Some references might be helpful.

Response: We appreciate this comment very much. We have clarified for the motivation and references in the Revision: “As described in the Introduction, our key question focused on object color knowledge effects derived from color-perception processes, which have been the main focus of previous studies (Goldberg et al., 2006; Hsu et al., 2012; Simmons et al., 2007). Following this tradition, the searchlight mapping was performed within a functionally-defined, VOTC-color-perception mask (**Fig 2B**, left panel; defined by contrasting chromatic stimuli to grayscale stimuli in a color perceptual localizer involving 14 healthy controls (Wang et al., 2020)). Within this VOTC color perception mask, the top 300 voxels (i.e., with the 300 highest r values) in the RSA results were chosen for each participant and then overlaid across all the healthy controls (N = 33).” (Results section, page 4).

Q6: Line 90-91 Is calcarine cortex a higher visual processing area than VOTC? Line 236-237 Same question for occipital pole.

Response: The calcarine and occipital pole are “earlier” and more primary than the VOTC in the visual hierarchy. We meant that they were “upstream” of the VOTC in visual processes. Given the potential confusion, we have revised the writing and used anatomical labels directly for clarity: “A recent study further revealed that object color knowledge deficits in SD was related to decreased structural connections between the left fusiform gyrus (spanning from the anterior to posterior ventral temporal lobe) and the early visual cortex (calcarine)” (Introduction section, page 3), and “it is still possible that the integrity was affected by lesions in the WM connections with the early visual cortex” (Results section, page 6). Thank you.

Q7: Line 186 What kind of word cues were used?

Response: Correct answers were provided. The wording has been revised to “For the objects that the participants failed to name or match, they were provided correct answers to ensure object recognition.” (Materials and Methods section, page 12). Thank you.

Other minor points where wording needs correction or further explication:
Q8: Lines 68-69 "origin of the potential modulation" is unclear

Response: Corrected to “A key challenge is that in a typical system, information structures derived from various levels of perception and language are difficult to disentangle (cat and dog are related both in visual and language spaces, and in tactile and auditory spaces).” (Introduction section, page 3).

Q9: Lines 72 - "fully depriving sensory experience" perhaps s/b "resulting in full deprivation of sensory experiences"

Response: Corrected. Thank you.

Q10: Line 81 "with necessity and/or sufficiency" should be reworded.

Response: Corrected to “Do such connections with the language system contribute to supporting object color knowledge behavior?” (Introduction section, page 3).

Q11: Line 81-82 Something is missing in "does VOTC the neural representation know". Maybe s/b "does the neural representation in VOTC "know"

Response: Corrected. Thank you.

Q12: Line 85 "but inconclusive" s/b "but this evidence is inconclusive"

Response: Deleted. Thank you.

Q13: Line 89 "atrophy is not confomative" Conformative with what?

Response: Corrected to "Given that brain atrophy in SD patients may affect the functionality of surrounding or remote areas” (Introduction section, page 3).

Q14: Line 437 Perhaps s/b "these results are in accord with previous results showing that these large WM bundles are Involved in supporting…"

Response: Corrected. Thank you.


Reviewer #2: This is a study on 33 stroke survivors and 35 age-matched controls. Participants performed fMRI experiments of object color knowledge: they were shown greyscale pictures of familiar fruits and vegetables. They were asked to judge whether their typical skin color of the fruit/vegetable was red. White matter tracts were studied using diffusion MRI. The main result (as far as I understand it) is that the structural integrity of white matter connections between high-level visual cortex and the left anterior temporal lobe was causally related to object-color knowledge.

Q1: This study contains potentially important results, but in its current form it's difficult or sometimes even impossible to follow. In my opinion, the paper should be rewritten in a much more streamlined and clarified version. The new version should follow a clear experimental logic; important results should be properly highlighted. For example, if, as it seems, the main topic of the study is the brain organization of object-color knowledge, why is there no mention of color in the title, and color only appears midway in the abstract? This is confusing.

Response: We appreciate this comment. We have thoroughly revised the manuscript for clarity. Major changes include the following: 1) clarifying the question framing, patient selection rationale, and corresponding analyses schemes in the Introduction; 2) organizing the Result sections into two main lines of analyses (WM tract integrity effect on VOTC neural representation; WM tract integrity effect on behavior); 3) highlighting important results, and moving methodological details into the Materials and Methods section; 4) clarifying the design rationales for the behavior tasks in the scanner (fMRI experiments) and the behavioral assessments outside the scanner; 5) explicitly stating in the Abstract that color knowledge was used as a test case for probing object knowledge representation; and 6) hiring a professional editor to proofread the paper. We believe that the clarity of the paper has been improved, and we thank the reviewer for this important suggestion.

Other points:
Q2: Abstract: I don't see any direct link between the study results and "embodied object knowledge neural representation."

Response: We used this term loosely to refer to the neural overlap between knowledge and perception systems (Goldberg et al., 2006; Hsu et al., 2012; Simmons et al., 2007). We agree with the reviewer that without a direct test of the perceptual process, using this term is counterproductive. We have removed this term in the revised manuscript. Thank you.

Q3: The hemispheric side of lesion is obviously highly relevant in this study on the role of language in color knowledge, but it is not even clear from the outset how many of the 33 patients had left hemisphere lesions.

Response: Thank you for the suggestion. We have added the hemispheric-side lesion information to the Abstract of the revised manuscript as follows: “(N = 33; 18 with left-hemisphere lesions, 11 with right-hemisphere lesions, and 4 with bilateral lesions)”, in addition to the information provided in **Table 1**.

Notably, when only patients with left hemisphere lesions (N=18) were considered, the key analyses revealed a pattern comparable to that of the whole group. We have added this information to the revised manuscript: “Finally, when patients with only left hemisphere lesions (N=18) were considered, the key analyses revealed a pattern comparable to that of the whole group, although not reaching statistical significance for the behavioral analyses due to reduced N (partial rho value between VOTC-LdlATL connection integrity and VOTC neural RSA strength = 0.53, *p* = 0.03; between VOTC-LdlATL connection integrity and object color knowledge behavior score = 0.43, *p* = 0.09).” (Results section, page 9).

Q4: Line 120. "typical lesion distribution for a stroke population." Indeed, most patients seem to have lesions in the middle cerebral artery territory, which is indeed the most frequent stroke localization. However, the most informative lesion sites to test the authors' hypotheses on VOTC are in the territory of the posterior cerebral artery.

Response: Thank you for this important question, which prompted us to clarify the motivation and rationale for patient inclusion and the framing of key questions. We wish to clarify that the key question is the role of the VOTC-language connection—whether it modulates the VOTC’s object knowledge representation, and whether it contributes to supporting object color knowledge behavior. To this end, we selected patients whose lesions affected the connection of interest, and not the VOTC. This approach allowed us to test the effects of the connection on VOTC functionality. Indeed, when testing whether the VOTC and its connection with language each contribute to behavior, such patient selection is not optimal for observing VOTC signals. In the revised manuscript, we have clarified the rationale for patient inclusion (focusing on the role of the connection) and the corresponding discussion for interpreting the negative results regarding VOTC RSA strength and behavior:

“Specifically, we tested whether lesions affecting the connection between the VOTC and language systems affect the local neuro-functional strength of object color representation in the VOTC (Analyses series 1) and whether the connection contributes to supporting object color knowledge behavior (Analyses series 2). We therefore deliberately included stroke patients suffering from lesions outside the VOTC to minimize the confounding effects of local damage to the VOTC on its functionality.” (Introduction section, page 4).

“Note that while the VOTC functional integrity was significantly modulated by its connection with the LdlATL, its correlation with object color behavior was not significant. This null effect should be interpreted with caution. As we deliberately selected stroke patients whose lesions largely spared the VOTC, this patient cohort may not be optimal for revealing the effects of the VOTC. …The key point is that, in explaining behavior, the effects of the VOTC-LdlATL connection are beyond the potential effects of VOTC functionality.” (Discussion section, page 10).

Q5: Line 90. "the object knowledge deficits might still be attributable to the VOTC's decreased structural connection between the left fusiform gyrus and calcarine rather than anterior integrity." But this would be highly unlikely because patients with cortical blindness from bilateral V1 lesions or with cerebral achromatopsia can show perfectly normal color knowledge (see, e.g., Bartolomeo, P., Liu, J., & Spagna, A. (2024). Colors in the mind's eye. _Cortex_, _170_, 26-31).

Response: We appreciate the advice to better discuss the relationships among several lines of literature findings. Indeed, the previous description of Chen et al. (2020) was not precise enough, which caused confusion.

Chen and colleagues reported that the white matter connection (measured by axial diffusivity) between the left fusiform gyrus and the calcarine in 33 SD patients was significantly correlated with object color knowledge behaviors (partial r = -0.56, *p* = 0.002). The left fusiform gyrus in that analysis was taken from the AAL template, including not only regions overlapping with our VOTC-color-perception mask but also regions spanning more anteriorly. We are not sure what part of the connection along that pathway contributes to the deficits. Considering the normal color knowledge profiles of patients with cortical blindness from bilateral V1 lesions or with cerebral achromatopsia, the deficits in those SD patients may be attributable to any point along the ventral pathway beyond the V1 and/or color perception regions affected in patients with achromatopsia. We have added relevant references and made revisions accordingly:

“This anterior non-sensory color knowledge representation thus constitutes a strong candidate that may support normal object color knowledge in individuals with congenital blindness, or patients with cortical blindness from bilateral V1 lesions or with cerebral achromatopsia (Bartolomeo et al., 1998; Bartolomeo et al., 2024; Wang et al., 2020).” (Introduction section, page 3).

“A recent study further revealed that object color knowledge deficits in SD were related to decreased structural connections between the left fusiform gyrus (spanning from the anterior to posterior ventral temporal lobe) and the early visual cortex (calcarine). Given that brain atrophy in SD patients may affect the functionality of surrounding or remote areas, it is unclear what aspects of this connection in relation to the VOTC account for the object color knowledge deficits in these patients.” (Introduction section, page 3).

Q6: Line 152. "The group-level whole-brain analyses in the healthy controls revealed the right lingual gyrus as the top cluster representing object color knowledge." This result looks really implausible. It is simply false that this finding is, as the authors state, "in line with those reported in the literature" (line 154). On the contrary, abundant evidence points to the *left* VTC. See, eg, one of the studies cited in the present paper: "We observed increased activation during both perception and memory retrieval of chromatic compared to achromatic stimuli in overlapping areas of the *left* lingual gyrus" (Hsu et al, 2012). Another cited study (Wang et al 2020) discusses "object-color-knowledge representation effects in the *left* ATL." The right hemisphere result might be an artifact of co-variating for object shape RDM: the authors are testing for object colors, not abstract colors. Object-color knowledge probably requires the integration of object shape and color in the brain. Thus, controlling for object shape can lead to artifactual results. More generally, the authors should be much more careful when citing the existing literature. I didn't check all the other citations.

Response: Thank you for this very important comment. We have corrected the specific citations for which we overlooked the lateralization information, and rigorously examined all the references in the manuscript.

We have added a table (**S7 Table**) summarizing the results of the object color knowledge studies in the literature. The studies that used word stimuli tended to observe left hemisphere activation, whereas those that used picture stimuli tended to observe right-hemisphere activation. The right hemisphere tendency in the individualized and group results in our study (as our stimuli were grey-scale pictures) aligned with this trend. We have revised the text and citations as follows: “This functional mask is largely in line with those reported in previous studies about object color knowledge, especially those using picture stimuli (Bannert and Bartels, 2013; Chao and Martin, 1999; Martin et al., 1995; Rich et al., 2006; Takashima et al., 2024; Vandenbroucke et al., 2016; see **S7 Table**).” (Results section, page 4).

Your point that “the right hemisphere result might be an artifact of co-variating for object shape RDM” is well taken. Yet when we directly correlated the neural RDMs with the color-similarity RDM, without regressing out the other variables including shape, the whole-brain analysis still revealed similar activation in the right lingual gyrus (see **S1A Fig**, which has been added to the Supporting information).

We have thoroughly checked the citations throughout, and again, we thank you for bringing this to our attention.

Q7: Line 187: Patients had similar performance accuracy (0.90) and SD (0.09) as controls (0.92 ± 0.06) on the color knowledge test. How can their lesion or WM pattern be informative on the neural bases of color knowledge?

Response: These behavioral results were from the task fMRI experiment performed *inside the scanner*, which was a tailored “easy” version, selecting those items that the subjects knew. This was done to optimally measure meaningful VOTC neural activity for color knowledge. Thus, the behavioral performance for these *inside the scanner* task was at the ceiling for both groups. A separate set of neuropsychological tests was administered outside the scanner to measure object color knowledge performance properly. On these outside-scanner neuropsychological tests, the patients did show deficits (**Fig 3**), which were used for the brain-behavior correlation analyses.

This question made us realize that our original description of the behavioral methods was not clear enough (see a similar question by **Reviewer 3, Q3**). We have carefully revised the sections related to the behavioral results in the Materials and Methods, Results, and Figures accordingly. 1) In the Materials and Methods section, we rewrote and reorganized the “task-fMRI experiment” (page 12), “Neuropsychological tests” (page 13), and “Object color behavior in patients: Underlying neural correlates and specificity tests” (page 17) sections to clarify the behavioral procedures and the related rationale. 2) In the Results section, where this behavioral result was described, we moved some details to the Materials and Methods section and explicitly stated that the comparably at-ceiling behavioral performances were in-scanner tests (page 5). And 3) to prevent confusion, the Figures (**Fig 3**) now show the behavioral performances in the neuropsychological tests, with the in-scanner tailored easy version described in the text.

Q8: Line 483. "Thirty-three stroke patients (25 males), without major posterior lesions". This exclusion criterion needs to be motivated. By the way, what does "posterior" mean here anatomically? Postrolandic?

Response: Thank you for pointing this out. Motivation and specification have been clarified in the Revision: “Specifically, we tested whether lesions affecting the connection between the VOTC and language systems affect the local neuro-functional strength of object color representation in the VOTC (Analyses series 1) and whether the connection contributes to supporting object color knowledge behavior (Analyses series 2). We therefore deliberately included stroke patients suffering from lesions outside the VOTC to minimize the confounding effects of local damage to the VOTC on its functionality.” (Introduction section, page 4; see also response to **Q4** above). Similar specification has now been added in the Materials and Methods section: “Thirty-three stroke patients (25 males), without major lesions affecting the VOTC, were recruited from the First Hospital of Shanxi Medical University. …lesion location involving the cortex and/or subcortical WM and not the VOTC” (page 12).

Minor points
Q9: The English is often incorrect and should be reviewed. E.g., in the abstract: "in a group of patients suffered from stroke" should be "having suffered from" or something similar.

Response: Corrected. We have also hired a professional editor who is a native speaker of English to proofread the revised manuscript. Thank you for the suggestion.

Q10: Line 89. What does "atrophy is not conformative" mean?

Response: Corrected to “Given that brain atrophy in SD patients may affect the functionality of surrounding or remote areas” (Introduction section, page 3).

Reviewer #3: The question of the relationship between language and cognition is an old and perentially interesting one. Recent work by Fedorenko et al., has argued in favor for the modularity of language from the rest of cognition based on language localizer results. The study here is a fascinating counterargument, showing neural evidence in favor of linguistic modulation of ventral occipitotemporal cortex (VOTC) that has been previously linked to knowledge of object color (and perhaps visual knowledge more generally).

This is a valuable addition to the literature.
I have two main questions/comments:
Q1: First, I wish the authors made it a bit clearer what they think the language regions are doing. How are they modulating VOTC? As a source of ideas — and I don't make a habit of citing myself in reviews — please see:
Lupyan, G. (2009). Extracommunicative Functions of Language: Verbal Interference Causes Selective Categorization Impairments. *Psychonomic Bulletin & Review*, *16*(4), 711-718. ~[https://doi.org/10.3758/PBR.16.4.711](https://doi.org/10.3758/PBR.16.4.711)~
and
Lupyan, G., Mirman, D., Hamilton, R. H., & Thompson-Schill, S. L. (2012). Categorization is modulated by transcranial direct current stimulation over left prefrontal cortex. *Cognition*, *124*(1), 36-49. ~[https://doi.org/10.1016/j.cognition.2012.04.002](https://doi.org/10.1016/j.cognition.2012.04.002)~
(as well as the citations to older literature, e.g., Cohen, Kelter, and other members of the Konztanz group — cited in the lit review)

Response: We very much appreciate these highly relevant references. We think this provides a very nice possible explanation for our findings, and have added the following text to the Discussion: “Another, non-mutually exclusive possibility, is that language representations play an online modulatory role in categorizing object properties, especially along perceptual dimensions such as color, where other differences needed to be abstracted away (Lupyan, 2009; Lupyan et al., 2012)” (page 11). (We have also been very much inspired by your discussions in other contexts related to this broad issue).

Q2: Second, I am surprised at the variability of the performance of the control group (Fig 3A). Can you say something abotu what predicts the *control* group performance? I understand that the control group served as a baseline for identifying tractographic norms against which the patients were compared, but is there a way to use the tractography analyses in the contrl group to see if any differences in the connection between language regions and VOTC predict *their* performance?

Response: The variability in the performance of the control group is because the control participants, who were selected to be demographically matched with the patients, spanned a wide range of ages (from 31 to 65 years old) and years of education (from 7 to 16). As suggested, we carried out exploratory Spearman’s correlation analyses between the mean FA value of the tracts connecting the VOTC and left language regions and the object color composite score across the healthy controls (n = 34). Only the FA value of the VOTC-LdlATL tract was significantly positively correlated with the object color composite score.

We have added this additional convergent result to the Results section: “Finally, given that the healthy control participants were selected to be demographically matched with the patients, they spanned a wide range of ages (from 31 to 65 years) and years of education (from 7 to 16), and showed decent variability in the neuropsychological test performance. Therefore, we carried out exploratory Spearman’s correlation analyses between the mean FA value of the tracts connecting the VOTC and left language regions and the object color composite score across the healthy controls (n = 34). Only the FA value of the VOTC-LdlATL tract was significantly positively correlated with the object color composite score (VOTC-LdlATL: partial rho = 0.42, *p* = 0.01; other tracts: partial rhos < 0.31, *p*s > 0.07). These results converge with the patient findings and highlight the role of the VOTC-LdlATL tract in supporting object color knowledge behaviors.” (pages 7-8). Thank you!

Other comments:
Q3: The description of the behavioral methods make it somewhat hard to connect the methods to the relevant figures, to the relevant results. For example, it's not clear what the numeric results are in the task shown in **Fig. 2****A** (and it's not clear what it's called, making it difficult to find the relevant bit of methods).

Response: We very much appreciate this comment. A related question was raised by **Reviewer 2, Q7**. Please see our detailed responses to that comment. Briefly here, in the revised manuscript, we have updated the sections related to behavioral results in the Materials and Methods, Results, and Figures to avoid confusion. Moreover, we have changed the names of the object color behavioral tasks from “verbal object color task” and “non-verbal object color task” to “grayscale picture-color word matching task (verbal)” and “object color true/false judgment task (non-verbal)”, respectively, and we have explicitly stated these behavioral tasks when the relevant results were presented in the Materials and Methods, Results and Fig 3.

Please proofread for grammar - there are many places with incorrect number agreement (e.g., ) and other typos, e.g., "comes from patient studies, but inconclusive"

Response: Corrected. We have also hired a professional editor who is a native speaker of English to proofread the revised manuscript. Thank you for the suggestion.

-Gary Lupyan

**References**

Bannert, M.M., and Bartels, A. (2013). Decoding the yellow of a gray banana. Current Biology *23*, 2268-2272.

Bartolomeo, P., Bachoud-Lévi, A.-C., De Gelder, B., Denes, G., Dalla Barba, G., Brugières, P., and Degos, J.-D. (1998). Multiple-domain dissociation between impaired visual perception and preserved mental imagery in a patient with bilateral extrastriate lesions. Neuropsychologia *36*, 239-249.

Bartolomeo, P., Liu, J., and Spagna, A. (2024). Colors in the mind’s eye. Cortex *170*, 26-31.

Chao, L.L., and Martin, A. (1999). Cortical regions associated with perceiving, naming, and knowing about colors. Journal of cognitive neuroscience *11*, 25-35.

Goldberg, R.F., Perfetti, C.A., and Schneider, W. (2006). Perceptual knowledge retrieval activates sensory brain regions. Journal of Neuroscience *26*, 4917-4921.

Hsu, N.S., Frankland, S.M., and Thompson-Schill, S.L. (2012). Chromaticity of color perception and object color knowledge. Neuropsychologia *50*, 327-333.

Lupyan, G. (2009). Extracommunicative functions of language: Verbal interference causes selective categorization impairments. Psychonomic bulletin & review *16*, 711-718.

Lupyan, G., Mirman, D., Hamilton, R., and Thompson-Schill, S.L. (2012). Categorization is modulated by transcranial direct current stimulation over left prefrontal cortex. Cognition *124*, 36-49.

Martin, A., Haxby, J.V., Lalonde, F.M., Wiggs, C.L., and Ungerleider, L.G. (1995). Discrete cortical regions associated with knowledge of color and knowledge of action. Science *270*, 102-105.

Rich, A.N., Williams, M.A., Puce, A., Syngeniotis, A., Howard, M.A., McGlone, F., and Mattingley, J.B. (2006). Neural correlates of imagined and synaesthetic colours. Neuropsychologia *44*, 2918-2925.

Simmons, W.K., Ramjee, V., Beauchamp, M.S., McRae, K., Martin, A., and Barsalou, L.W. (2007). A common neural substrate for perceiving and knowing about color. Neuropsychologia *45*, 2802-2810.

Takashima, A., Carota, F., Schoots, V., Redmann, A., Jehee, J., and Indefrey, P. (2024). Tomatoes Are Red: The Perception of Achromatic Objects Elicits Retrieval of Associated Color Knowledge. Journal of Cognitive Neuroscience *36*, 24-45.

Vandenbroucke, A.R., Fahrenfort, J., Meuwese, J., Scholte, H., and Lamme, V. (2016). Prior knowledge about objects determines neural color representation in human visual cortex. Cerebral cortex *26*, 1401-1408.

Wang, X., Men, W., Gao, J., Caramazza, A., and Bi, Y. (2020). Two forms of knowledge representations in the human brain. Neuron *107*, 383-393. e385.
