# Supplementary material for: Object knowledge representation in the human visual cortex requires a connection with the language system
Source: PLoS Biol. 2025 May 20;23(5):e3003161. doi: 10.1371/journal.pbio.3003161 (PMC12091770; doi:10.1371/journal.pbio.3003161)
Supplement: Attachment — Submitted filename: response-to-review file.docx [file pbio.3003161.s019.docx]

We greatly appreciate the constructive comments and suggestions from the Reviewers. We have carefully revised the manuscript accordingly to the Reviewers’ remaining suggestions. Below we explain how each comment was addressed.

Reviewer #2: The authors should be commended for revising their paper extensively, rendering it more readable.
I'm still uncertain about the anatomy, particularly how lesions in the MCA territory could affect the white matter tracts identified as critical for color knowledge. Given that ILF and IFOF are supplied by the PCA, it remains unclear how damage within the MCA territory would disrupt these pathways. Perhaps a disconnectome analysis (see https://rdcu.be/ecVVX) could help clarify this issue.

Response: We appreciate this comment very much, which helps us to clarify the anatomy of the lesion distribution in our study and the rationale for using FA values for the main analyses. First, we have clarified in the Revision (page 4) that while the patient group as a whole follows the typical lesion distribution in the MCA territory, other parts of the brain were also affected in some cases (e.g., 8 out of the 33 patients also had lesions in the posterior cerebral artery territory). Second, the point raised by the Reviewer is exactly why we focused on the FA values and we have made this explanation in the Revision (page 5): Past studies, including the reference suggested by the Reviewer, have consistently documented that stroke-related damage may extend beyond visible lesions due to multiple mechanisms (Galantucci et al., 2011; Ivanova et al., 2016; Thiebaut de Schotten et al., 2020; Yuan et al., 2017). We thus focused on the fractional anisotropy (FA), which, by measuring the directional constraint of water diffusion, is commonly considered to be a sensitive measure of the structural integrity of WM fibers.

The authors conclude the abstract by stating that their findings provide "novel anatomical and functional targets for the rehabilitation of knowledge loss." However, I find this claim and its elaboration in the discussion too vague. How exactly could knowledge loss be restored by targeting these regions? Unless the authors have something specific in mind, I would suggest removing the mention of rehabilitation.

Response: Thank you. In light of this comment, we have removed the statement “and offering novel anatomical and functional targets for the rehabilitation of knowledge loss” from the Abstract in the revised manuscript.

In the revised Discussion section, we have elaborated on this point as follows: “These findings, by highlighting the role of perception-language system connections in knowledge loss, motivate rehabilitation strategies that target such components, such as cognitive trainings that involve vision-language-alignment and/or brain stimulation targeting the underlying connectional functionality.” (page 11)

Reviewer #3: The reviewers did a good job addressing my earlier concerns. In my estimation I think they also adequately addressed the (somewhat more critical) reviews of the other referees. My one remaining suggestion is to make the behavioral results a bit more salient, e.g., please add the means to the Fig 3A plots and make the control-patient inferential test more prominent in the text. Which contrasts show significant differences?

Response: We appreciate the comment very much and have clarified the description of behavioral results in the text and in the Fig 3A plots in the Revision following the reviewer’s suggestion. Specifically, we clarified that the raw accuracies are shown in the Fig 3A dot plots. We also clarified that we focused on case analyses, obtaining patients’ standardized scores based on the single-case Bayesian test for deficit for the subsequent brain-behavior correlation analyses. We did not report direct contrasts between healthy controls and patients at the group level because such comparisons may not be meaningful due to the heterogeneous nature of each group.

The revised Results section is as follows, “**Fig 3A** (bottom panel) shows the raw accuracies of these neuropsychological tests for each of the healthy controls and stroke patients (see **S2 Table** for the detailed behavioral scores of each patient in each task). Each patient’s standardized scores of these tests were computed as the point estimates of effect sizes based on the single-case Bayesian test for deficit, which controls for the variation in demographic factors (i.e., age, gender, and years of education) among the two groups.” (page 7)

We also updated the caption of Fig. 3A as follows, “Dot plots show raw accuracies of each participant (bars denotes mean values ± 1 standard error)”. (page 25)

**Reference**

Galantucci, S., Tartaglia, M.C., Wilson, S.M., Henry, M.L., Filippi, M., Agosta, F., Dronkers, N.F., Henry, R.G., Ogar, J.M., and Miller, B.L. (2011). White matter damage in primary progressive aphasias: a diffusion tensor tractography study. Brain *134*, 3011-3029.

Ivanova, M.V., Isaev, D.Y., Dragoy, O.V., Akinina, Y.S., Petrushevskiy, A.G., Fedina, O.N., Shklovsky, V.M., and Dronkers, N.F. (2016). Diffusion-tensor imaging of major white matter tracts and their role in language processing in aphasia. Cortex *85*, 165-181.

Thiebaut de Schotten, M., Foulon, C., and Nachev, P. (2020). Brain disconnections link structural connectivity with function and behaviour. Nature communications *11*, 5094.

Yuan, B., Fang, Y., Han, Z., Song, L., He, Y., and Bi, Y. (2017). Brain hubs in lesion models: Predicting functional network topology with lesion patterns in patients. Scientific Reports *7*, 17908.
